# Supplementary material for: Functional movement disorder is associated with abnormal interoceptive brain activity: a task-based functional MRI study
Source: Front Psychiatry. 2025 Feb 21;16:1473913. doi: 10.3389/fpsyt.2025.1473913 (PMC11885509; doi:10.3389/fpsyt.2025.1473913)
Supplement: Supplementary file 1 [file DataSheet1.docx]

SUPPLEMENTARY INFORMATION

fMRI pre-processing

Preprocessing of fMRI data was performed using AFNI (v7.12; http://afni.nimh.nih.gov/afni). Anatomical images were spatially transformed to the AFNI standard Talairach space (TT_N27_SSW.nii.gz) using the @SSwarper function. Preprocessing of the three runs of task were analyzed together using afni_proc.py.

Multi-echo data were combined using the tedana method (Kundu et al., 2012). The first two volumes of each voxel’s time course were excluded from analysis to allow the fMRI signal to reach steady state. We set motion limit at 0.3 and identifying volumes with more than 10% of outliers as defined with 3dToutcount tool in AFNI in a censor file to be used subsequently in the regression analysis. Motion correction and spatial transformation were implemented in a single image transformation. The EPI data were smoothed using a 4 mm full-width at half-maximum Gaussian kernel and using the in-mask option.

Maps for each condition and subject were created using the following script:

*preprocess_taskFMRI_02b_TT27.sh*

*#!/usr/bin/bash*

*#preprocess_taskFMRI_02b_TT27.sh*

*#interoception project, 07N0190*

*#uses afni_proc.py to preprocess the task sequences (and SSwarper to prepare mprage for afni_proc.py)*

*#this is an alternate version that uses the TT27 template instead of the MNI template*

*export OMP_NUM_THREADS=16*

*dataDir=/raids/newhmcsraid2/07N0190_FMD/interoception/data*

*outDir=/raids/newhmcsraid2/07N0190_FMD/interoception/data_proc*

*scriptDir=/raids/newhmcsraid2/07N0190_FMD/interoception/scripts*

*template=/usr/local/afni/TT_N27_SSW.nii.gz*

*echoes="14.5 32.3 50.1"*

*mkdir ${outDir}*

*#for sj in FMD0001 FMD0002 FMD0003 FMD0004 FMD0005 FMD0006 FMD0007 FMD0008 FMD0009 FMD0010 FMD0011 FMD0012 FMD0013 FMD0014 FMD0015 FMD0016 HV0002 HV0004 HV0007 HV0008 HV0009 HV0010 HV0011*

*for sj in HV0001 HV0014 HV0015*

*do*

*mkdir ${outDir}/${sj}*

*echo Sourcing data from ${dataDir}/${sj}*

*echo Saving results to ${outDir}/${sj}*

*if [ -f ${outDir}/${sj}/mprage_TT27/anatQQ.mprage.nii ]; then*

*echo "SSwarper already run"*

*else*

*mkdir ${outDir}/${sj}/mprage_TT27*

*echo Running SSwarper for ${sj}*

*/raids/hmcsraid3/parker/@SSwarper -input ${dataDir}/${sj}/mprage/mprage.nii -base ${template} -subid mprage -omp 16 -odir ${outDir}/${sj}/mprage_TT27*

*fi*

*#preprocess all task runs together*

*echo Running afni_proc.py for ${sj} all task runs*

*mkdir ${outDir}/${sj}/task_TT27*

*cd ${outDir}/${sj}/task_TT27*

*afni_proc.py -subj_id task_TT27 \*

*-blocks despike tshift align tlrc volreg mask combine blur scale regress \*

*-copy_anat ${outDir}/${sj}/mprage_TT27/anatSS.mprage.nii \*

*-anat_has_skull no \*

*-dsets_me_run ${dataDir}/${sj}/taskA/e*.nii \*

*-dsets_me_run ${dataDir}/${sj}/taskB/e*.nii \*

*-dsets_me_run ${dataDir}/${sj}/taskC/e*.nii \*

*-echo_times ${echoes} \*

*-reg_echo 2 \*

*-tcat_remove_first_trs 2 \*

*-align_opts_aea -cost lpc+ZZ \*

*-tlrc_base ${template} \*

*-tlrc_NL_warp \*

*-tlrc_NL_warped_dsets \*

*${outDir}/${sj}/mprage_TT27/anatQQ.mprage.nii \*

*${outDir}/${sj}/mprage_TT27/anatQQ.mprage.aff12.1D \*

*${outDir}/${sj}/mprage_TT27/anatQQ.mprage_WARP.nii \*

*-volreg_align_to MIN_OUTLIER \*

*-volreg_align_e2a \*

*-volreg_tlrc_warp \*

*-mask_epi_anat yes \*

*-combine_method tedana \*

*-combine_tedort_reject_midk no \*

*-blur_size 4 \*

*-blur_in_mask yes \*

*-regress_motion_per_run \*

*-regress_censor_motion 0.3 \*

*-regress_censor_outliers 0.1 \*

*-regress_apply_mot_types demean deriv \*

*-regress_est_blur_epits \*

*-regress_basis 'BLOCK(10,1)' \*

*-regress_stim_times \*

*${dataDir}/${sj}/stim_files/body_stim_times.txt \*

*${dataDir}/${sj}/stim_files/stomach_stim_times.txt \*

*${dataDir}/${sj}/stim_files/heart_stim_times.txt \*

*${dataDir}/${sj}/stim_files/target_stim_times.txt \*

*-regress_stim_labels body stomach heart target \*

*-regress_stim_times_offset -5 \*

*-html_review_style basic*

*python ${scriptDir}/afni_proc_mod.py proc.task_TT27*

*tcsh -xef proc.task_TT27_mod |& tee output.proc.task_TT27_mod*

*cd ${outDir}/${sj}/task_TT27/task_TT27.results*

*python2.7 /usr/local/afni/apqc_make_tcsh.py -review_style basic -subj_dir . -uvar_json out.ss_review_uvars.json*

*tcsh @ss_review_html |& tee out.review_html*

*python2.7 /usr/local/afni/apqc_make_html.py -qc_dir QC_task_TT27*

Each interoception condition (heart, stomach, body) was then contrasted against the exteroception (target) and the resulting aps enter for a group analysis using a multivariate model implemented with the AFNI function 3dMVM (Chen et al., 2014) as described in the script *MVM_Target_command.txt*.

Script:

*dMVM -prefix MVM_results_Target -jobs 24 \*

*-mask Mask3mm.nii \*

*-bsVars Group \*

*-wsVars Stim \*

*-num_glt 12 \*

*-gltLabel 1 BvsT_FMD -gltCode 1 'Group : 1*FMD Stim : 1*BvsT' \*

*-gltLabel 2 SvsT_FMD -gltCode 2 'Group : 1*FMD Stim : 1*SvsT' \*

*-gltLabel 3 HvsT_FMD -gltCode 3 'Group : 1*FMD Stim : 1*HvsT' \*

*-gltLabel 4 BvsOthers_FMD -gltCode 4 'Group : 1*FMD Stim : 1*BvsT -0.5*SvsT -0.5*HvsT' \*

*-gltLabel 5 BvsT_HV -gltCode 5 'Group : 1*HV Stim : 1*BvsT' \*

*-gltLabel 6 SvsT_HV -gltCode 6 'Group : 1*HV Stim : 1*SvsT' \*

*-gltLabel 7 HvsT_HV -gltCode 7 'Group : 1*HV Stim : 1*HvsT' \*

*-gltLabel 8 BvsOthers_HV -gltCode 8 'Group : 1*HV Stim : 1*BvsT -1*SvsT & 1*BvsT -1*HvsT' \*

*-gltLabel 9 BvsT_FMDvsHV -gltCode 9 'Group : 1*FMD -1*HV Stim : 1*BvsT' \*

*-gltLabel 10 SvsT_FMDvsHV -gltCode 10 'Group : 1*FMD -1*HV Stim : 1*SvsT' \*

*-gltLabel 11 HvsT_FMDvsHV -gltCode 11 'Group : 1*FMD -1*HV Stim : 1*HvsT' \*

*-gltLabel 12 BvsOthers_FMDvsHV -gltCode 12 'Group : 1*FMD -1*HV Stim : 1*BvsT -0.5*SvsT -0.5*HvsT' \*

*-num_glf 2 \*

*-glfLabel 1 BvsT_interact_FMDvsHV -glfCode 1 'Group : 1*FMD & 1*HV Stim : 1*BvsT ' \*

*-glfLabel 2 BvsOthers_interact_FMDvsHV -glfCode 2 'Group : 1*FMD & 1*HV Stim : 1*BvsT -1*SvsT & 1*BvsT -1*HvsT' \-dataTable \*

*Subj Group Stim InputFile \*

*FMD0001 FMD BvsT FMD0001B_T_Bmap.nii \*

*FMD0002 FMD BvsT FMD0002B_T_Bmap.nii \*

*FMD0004 FMD BvsT FMD0004B_T_Bmap.nii \*

*FMD0005 FMD BvsT FMD0005B_T_Bmap.nii \*

*FMD0006 FMD BvsT FMD0006B_T_Bmap.nii \*

*FMD0007 FMD BvsT FMD0007B_T_Bmap.nii \*

*FMD0008 FMD BvsT FMD0008B_T_Bmap.nii \*

*FMD0009 FMD BvsT FMD0009B_T_Bmap.nii \*

*FMD0010 FMD BvsT FMD0010B_T_Bmap.nii \*

*FMD0011 FMD BvsT FMD0011B_T_Bmap.nii \*

*FMD0013 FMD BvsT FMD0013B_T_Bmap.nii \*

*FMD0014 FMD BvsT FMD0014B_T_Bmap.nii \*

*FMD0016 FMD BvsT FMD0016B_T_Bmap.nii \*

*FMD0001 FMD SvsT FMD0001S_T_Bmap.nii \*

*FMD0002 FMD SvsT FMD0002S_T_Bmap.nii \*

*FMD0004 FMD SvsT FMD0004S_T_Bmap.nii \*

*FMD0005 FMD SvsT FMD0005S_T_Bmap.nii \*

*FMD0006 FMD SvsT FMD0006S_T_Bmap.nii \*

*FMD0007 FMD SvsT FMD0007S_T_Bmap.nii \*

*FMD0008 FMD SvsT FMD0008S_T_Bmap.nii \*

*FMD0009 FMD SvsT FMD0009S_T_Bmap.nii \*

*FMD0010 FMD SvsT FMD0010S_T_Bmap.nii \*

*FMD0011 FMD SvsT FMD0011S_T_Bmap.nii \*

*FMD0013 FMD SvsT FMD0013S_T_Bmap.nii \*

*FMD0014 FMD SvsT FMD0014S_T_Bmap.nii \*

*FMD0016 FMD SvsT FMD0016S_T_Bmap.nii \*

*FMD0001 FMD HvsT FMD0001H_T_Bmap.nii \*

*FMD0002 FMD HvsT FMD0002H_T_Bmap.nii \*

*FMD0004 FMD HvsT FMD0004H_T_Bmap.nii \*

*FMD0005 FMD HvsT FMD0005H_T_Bmap.nii \*

*FMD0006 FMD HvsT FMD0006H_T_Bmap.nii \*

*FMD0007 FMD HvsT FMD0007H_T_Bmap.nii \*

*FMD0008 FMD HvsT FMD0008H_T_Bmap.nii \*

*FMD0009 FMD HvsT FMD0009H_T_Bmap.nii \*

*FMD0010 FMD HvsT FMD0010H_T_Bmap.nii \*

*FMD0011 FMD HvsT FMD0011H_T_Bmap.nii \*

*FMD0013 FMD HvsT FMD0013H_T_Bmap.nii \*

*FMD0014 FMD HvsT FMD0014H_T_Bmap.nii \*

*FMD0016 FMD HvsT FMD0016H_T_Bmap.nii \*

*HV0001 HV BvsT HV0001B_T_Bmap.nii \*

*HV0002 HV BvsT HV0002B_T_Bmap.nii \*

*HV0004 HV BvsT HV0004B_T_Bmap.nii \*

*HV0005 HV BvsT HV0005B_T_Bmap.nii \*

*HV0006 HV BvsT HV0006B_T_Bmap.nii \*

*HV0007 HV BvsT HV0007B_T_Bmap.nii \*

*HV0008 HV BvsT HV0008B_T_Bmap.nii \*

*HV0009 HV BvsT HV0009B_T_Bmap.nii \*

*HV0010 HV BvsT HV0010B_T_Bmap.nii \*

*HV0011 HV BvsT HV0011B_T_Bmap.nii \*

*HV0013 HV BvsT HV0013B_T_Bmap.nii \*

*HV0014 HV BvsT HV0014B_T_Bmap.nii \*

*HV0016 HV BvsT HV0016B_T_Bmap.nii \*

*HV0001 HV SvsT HV0001S_T_Bmap.nii \*

*HV0002 HV SvsT HV0002S_T_Bmap.nii \*

*HV0004 HV SvsT HV0004S_T_Bmap.nii \*

*HV0005 HV SvsT HV0005S_T_Bmap.nii \*

*HV0006 HV SvsT HV0006S_T_Bmap.nii \*

*HV0007 HV SvsT HV0007S_T_Bmap.nii \*

*HV0008 HV SvsT HV0008S_T_Bmap.nii \*

*HV0009 HV SvsT HV0009S_T_Bmap.nii \*

*HV0010 HV SvsT HV0010S_T_Bmap.nii \*

*HV0011 HV SvsT HV0011S_T_Bmap.nii \*

*HV0013 HV SvsT HV0013S_T_Bmap.nii \*

*HV0014 HV SvsT HV0014S_T_Bmap.nii \*

*HV0016 HV SvsT HV0016S_T_Bmap.nii \*

*HV0001 HV HvsT HV0001H_T_Bmap.nii \*

*HV0002 HV HvsT HV0002H_T_Bmap.nii \*

*HV0004 HV HvsT HV0004H_T_Bmap.nii \*

*HV0005 HV HvsT HV0005H_T_Bmap.nii \*

*HV0006 HV HvsT HV0006H_T_Bmap.nii \*

*HV0007 HV HvsT HV0007H_T_Bmap.nii \*

*HV0008 HV HvsT HV0008H_T_Bmap.nii \*

*HV0009 HV HvsT HV0009H_T_Bmap.nii \*

*HV0010 HV HvsT HV0010H_T_Bmap.nii \*

*HV0011 HV HvsT HV0011H_T_Bmap.nii \*

*HV0013 HV HvsT HV0013H_T_Bmap.nii \*

*HV0014 HV HvsT HV0014H_T_Bmap.nii \*

*HV0016 HV HvsT HV0016H_T_Bmap.nii*

**Supplementary Table 1.** Group-differences in the Multidimensional Scale of Interoceptive Awareness (MAIA) subscale scores between patients with FMD and healthy controls

AttnRegulation= attention regulation; EMAwareness = emotional awareness

**References**

Kundu, P., Inati, S. J., Evans, J. W., Luh, W.-M., & Bandettini, P. A. (2012). Differentiating BOLD and non-BOLD signals in fMRI time series using multi-echo EPI. Neuroimage, 60(3), 1759–1770. https://doi.org/10.1016/j.neuroimage.2011.12.028

Chen G, Adleman NE, Saad ZS, Leibenluft E, Cox RW. Applications of multivariate modeling to neuroimaging group analysis: a comprehensive alternative to univariate general linear model. Neuroimage. 2014 Oct 1;99:571-88. doi: 10.1016/j.neuroimage.2014.06.027
